# Editorial: Respiratory Management of Extremely Preterm Infants

**DOI:** 10.3389/fped.2021.756819

**Published:** 2021-09-17

**Authors:** Hasan Özkan, Saadet Arsan, Yuan Shi

**Affiliations:** ^1^Department of Neonatology, Dokuz Eylül University, Izmir, Turkey; ^2^Department of Neonatology, Ankara University, Ankara, Turkey; ^3^Department of Neonatology, Chongqing Medical University, Chongqing, China

**Keywords:** extremely preterm infant, apnea of prematurity, lung protective ventilation, high frequency ventilation, volume guarantee ventilation, non-invasive ventilation, neurally adjusted ventilation assist, acute respiratory distress sendrom

The USA Neonatal Research Network reports that 82% of extremely preterm infants receive mechanical ventilation during their NICU stay (1). The lungs of extremely preterm infants are structurally and biochemically immature and extremely vulnerable and susceptible to ventilator-induced lung injury (2). A variety of respiratory support techniques, ventilation modes, and strategies such as non-invasive respiratory support, volume targeted ventilation, high frequency ventilation with lung recruitment maneuvers and neurally adjusted ventilatory assist (NAVA) have been studied in preterm infants but high-quality evidence for respiratory management of extremely preterm infants from well-designed clinical studies are very scares (3–6). With this Research Topic, we aim to reach more scientific evidence for optimizing respiratory management of ELBW infants. We present a special collection of 14 articles contributing sound evidence for the key concepts outlined.

First of all, Maturana et al. emphasizes the importance of careful interpretation of the conclusions of systemic reviews. They point out the potential sources of bias such as, heterogeneity in included populations, interventions, control groups and outcomes which might not be detected by the statistical analyses used in very well-known systematic reviews. We agree with the authors and find their approach applaudable.

Premature infants experience frequent apneas and intermittent hypoxemia episodes due to their respiratory instability. There are two articles on preterm apnea in this collection. Du et al. once more present the efficacy and safety of caffeine citrate in the treatment of intermittent hypoxia and bradycardia episodes in preterm infants in their multicentric, prospective longitudinal open-label, single-arm study. On the other hand, Martin et al. present an interesting *in-vitro* study to analyze the necessary pressure intensity and frequency of various models of tactile stimulation applied to terminate preterm apnea episodes.

Respiratory distress syndrome is the leading cause of respiratory failure in preterm infants. Two major underlying pathophysiologic mechanisms are surfactant deficiency and structural immaturity of their lungs. There is limited evidence for genetic susceptibility for the development of RDS in preterm infants (7). Wang et al. have investigated the relationship between rs1059057 gene polymorphism of *SP-A1* and RDS in Mongolian very premature infants. Their findings do not support a relation between the gene polymorphism of *SP-A1* and the incidence of RDS.

In recent years, minimal invasive surfactant administration is a promising new therapy for extremely preterm infants with respiratory distress syndrome. Han et al. have conducted a multicentric randomized study in China and demonstrated that minimal invasive surfactant administration was not superior concerning the incidence of bronchopulmonary dysplasia, but it was associated with benefits in reducing the incidence of patent ductus arteriosus, which suggests less hemodynamic interference to the extremely/very low birth weight infants during the critical transition phase of physiological adaptation soon after birth.

Evidence from clinical trials indicates non-invasive ventilation has been shown to decrease the need for mechanical ventilation and reduce the risk of bronchopulmonary dysplasia. Shi et al. provide a comprehensive review of the non-invasive respiratory support for management of respiratory distress in extremely preterm infants. This article reviews respiratory management with current NIV support strategies in extremely preterm infants both in delivery room as well as in the NICU and discusses the evidence to support commonly used NIV modes including nasal continuous positive airway pressure (NCPAP), nasal intermittent positive pressure ventilation (NIPPV), bi-level positive pressure (BI-PAP), high flow nasal cannula (HFNC) and newer NIV strategies currently being studied including, nasal high frequency ventilation (NHFV) and non-invasive neutrally adjusted ventilatory assist (NIV-NAVA).

Ding et al. have compared the clinical effects of three ventilation modes, NCPAP, SNIPPV and sequential SNIPPV/NCPAP in the treatment of preterm infants with severe RDS after extubation. They demonstrate that compared to NCPAP alone, the sequential treatment reduces the failure rate of extubation and increases the success rate of withdrawal of non-invasive ventilation within 1 week without increasing the risk of developing complications such as BPD and ROP. In another study Chen et al. compare the clinical efficacy of heated, humidified high-flow nasal cannula (HHHFNC) and nasal continuous positive airway pressure Their study show that HHHFNC not only shortens the oxygen exposure time but also effectively reduces the incidence of nasal injury and NEC. Additionally, HHHFNC achieves a significant advance in the time to reach full enteral feeding and reduces the days of hospitalization.

Glaser et al. provide a fantastic mini-review including many recent trials focusing the evolution and success of non-invasive ventilatory support, alternative means of delivering surfactant, and sustained lung inflation.

Despite best efforts to maximize non-invasive support, most of extremely preterm infants still need mechanical ventilation (1, 6). The use of volume targeted ventilation (VTV), high-frequency ventilation (HFV) and lung protective strategies in extremely preterm infants have gained popularity in recent years because of their potential to improve outcomes (4, 5). Ganguly et al. present a review of the existing literature including systematic reviews and meta-analysis for these popular modes of ventilation. They conclude that the evidence support the use of volume targeted and high frequency ventilation to reduce ventilator-induced lung injury but existing studies are not powered to determine significant reductions in mortality or morbidity in ELBW babies. Tüzün et al. evaluates the optimal high frequency oscillatory ventilation with volume-guarantee (HFOV-VG) settings in premature infants with respiratory distress syndrome, using the open-lung strategy. Their findings indicate that optimal levels are dynamic and change instantly and individually. Rong et al. demonstrate a similar efficacy of neurally adjusted ventilatory assist ventilation (NAVA) to conventional ventilation in respiratory outcomes of very low birth weight preterms with evolving or established BPD.

Acute respiratory distress syndrome (ARDS) is a clinical condition characterized by acute diffuse inflammatory lung injury and surfactant catabolism leading to severe hypoxemia. The management of ARDS in newborns consist of lung-protective ventilation strategies and therapeutic agents to improve gas exchange (8). Deliloglu et al. report two preterms diagnosed as neonatal ARDS according to the Montreux criteria, who benefitted from intratracheal surfactant plus budesonide treatment.

In recent years, both preclinical and clinical research have proven the efficacy and safety of stem cells in treating and preventing lung injury (9). However, there are currently no randomized clinical trials (RCTs) investigating the use of autologous cord blood mononuclear cells (ACBMNC) for the prevention of BPD in premature infants. Ren et al. present their placebo-controlled randomized multricentric study protocol to evaluate the efficacy of ACBMNC infusion in prevention of BPD.

Although the evidence to guide respiratory support strategies remains incomplete, the evidence available from this collection may provide an opportunity for a better approach to respiratory management and may provide additional clues for minimizing the adverse respiratory outcomes in extremely preterm infants requiring respiratory support.

## Author Contributions

HÖ, SA, and YS contributed to the development of this Research Topic and the accompanying editorial, as well as writing and editing the editorial manuscript. All authors contributed to the article and approved the submitted version.

## Conflict of Interest

The authors declare that the research was conducted in the absence of any commercial or financial relationships that could be construed as a potential conflict of interest.

## Publisher's Note

All claims expressed in this article are solely those of the authors and do not necessarily represent those of their affiliated organizations, or those of the publisher, the editors and the reviewers. Any product that may be evaluated in this article, or claim that may be made by its manufacturer, is not guaranteed or endorsed by the publisher.
